# Examination of Hearing in a Rheumatoid Arthritis Population: Role of Extended-High-Frequency Audiometry in the Diagnosis of Subclinical Involvement

**DOI:** 10.1155/2016/5713283

**Published:** 2016-04-27

**Authors:** Mar Lasso de la Vega, Ithzel Maria Villarreal, Julio Lopez-Moya, Jose Ramon Garcia-Berrocal

**Affiliations:** ^1^“Severo Ochoa” University Hospital, 28911 Madrid, Spain; ^2^“Puerta de Hierro-Majadahonda” University Hospital, 28222 Madrid, Spain

## Abstract

*Objective*. The aim of this study is to analyze the high-frequency hearing levels in patients with rheumatoid arthritis and to determine the relationship between hearing loss, disease duration, and immunological parameters.* Materials and Methods.* A descriptive cross-sectional study including fifty-three patients with rheumatoid arthritis was performed. The control group consisted of 71 age- and sex-matched patients from the study population (consecutively recruited in Madrid “Area 9,” from January 2010 to February 2011). Both a pure tone audiometry and an extended-high-frequency audiometry were performed.* Results*. Extended-high-frequency audiometry diagnosed sensorineural hearing loss in 69.8% of the patients which exceeded the results obtained with pure tone audiometry (43% of the patients). This study found significant correlations in patients with sensorineural hearing loss related to age, sex, and serum anti-cardiolipin (aCL) antibody levels.* Conclusion.* Sensorineural hearing loss must be considered within the clinical context of rheumatoid arthritis. Our results demonstrated that an extended-high-frequency audiometry is a useful audiological test that must be performed within the diagnostic and follow-up testing of patients with rheumatoid arthritis, providing further insight into a disease-modifying treatment or a hearing loss preventive treatment.

## 1. Introduction

Autoimmune hearing loss was reported for the first time by McCabe in 1979. [1–3] A description of a series of patients with bilateral progressive sensorineural hearing loss (SNHL) and altered immunological tests in response to an immunosuppressive treatment was performed.

Rheumatoid arthritis (RA) is a connective tissue disease that has a disseminated erosive arthropathy associated with systemic manifestations. This immune-mediated disease may entail mainly SNHL [4–11], which is to occur in 25.2% to 60% [5, 9, 12–14] of the cases. The incidence of conductive hearing loss in RA is estimated at 4.8% to 14% [4, 14–20].

The pathogenesis of SNHL in RA still remains unclear, although it is potentially related to vasculitis [21], neuritis, ototoxicity [12, 21, 22], or an immunological disorder [13]. Several studies have attempted to demonstrate this pathogenesis due to the existence of specific antigens in the inner ear [7, 10, 12, 23, 24]. The pathogenesis of conductive hearing loss is also unknown, although a number of studies have proposed that it originates from a disorder in the incudostapedial joint [4, 14–20].

This study aimed to assess the real prevalence of SNHL in RA using advanced audiometric analysis based on extended-high-frequency audiometry (HFA), thus considering its effectiveness and clinical utility for inclusion in the routine diagnostic hearing tests. Furthermore, we analyzed the immunological parameters to obtain an indicator of the inner ear impairment in these patients.

## 2. Materials and Methods

A cross-sectional descriptive study was carried out with comparative cases and controls matched for age and sex.

*Inclusion Criteria*. Fifty-three patients with RA (106 ears), both sexes, aged between 20 and 60, were diagnosed and treated in the office of the Rheumatology and Nephrology Departments of “Severo Ochoa” Hospital (Madrid, Spain). The diagnostic criteria and their respective scoring used for a definitive RA diagnosis were as follows: (A) joint involvement: 1 large joint = 0; 2–10 large joints = 1; 1–3 small joints (with or without involvement of large joints) = 2; 4–10 small joints (with or without involvement of large joints) = 3; >10 joints (at least 1 small joint) = 5; (B) serology (at least 1 test result is needed for classification): Negative Rheumatoid Factor (RF) and negative anti-citrullinated protein antibody (ACPA) = 0; low-positive RF or low-positive ACPA = 2; high-positive RF or high-positive ACPA = 3; (C) acute phase reactants (at least 1 test result is needed for classification): normal C-reactive protein (CRP) and normal Erythrocyte Sedimentation Rate (ESR) = 0; abnormal CRP or abnormal ESR = 1; (D) duration of symptoms: <6 weeks = 0; >6 weeks = 1. The classification criteria from the American College of Rheumatology (ACR) for RA (score-based algorithm: add score of categories A–D) consider that a score of > or = 6/10 is needed for classification of a patient as having a definitive RA.

Both a medical history and an ENT examination were performed, using the following* inclusion criteria*: patients who suffered from rheumatoid arthritis, both sexes, aged between 20 and 60.

*Exclusion Criteria*. Exclusion criteria are patients who suffered from another immune-mediated disease, inner ear disease, traumatic brain injury, Menière's disease, metabolic diseases, and cardiovascular disease, exposure to noise, and the use of ototoxic medication different from that used in the treatment of RA.

A control group of 71 patients (142 ears) was consecutively recruited in “Area 9” of Madrid, from January 2010 to February 2011.

The control group was composed of patients with a normal state of health, free from all signs or symptoms of ear disease and from obstructing wax in the ear canal, and with no history of exposure to noise, potentially ototoxic drugs, or familial hearing loss that attended our ENT department for head and neck or rhinologic disorders.

If the control population presented hearing thresholds in the limits of the normal range, according to the range of age and sex, assessed by pure tone audiometry (PTA) using frequencies of 125 to 8000 Hz, consequently an extended-high-frequency audiometry (HFA) of 8000 to 18000 Hz was also performed to determine the normal hearing parameters and use those parameters as references for the study.

A number of variables have been studied, such as demographic variables (age and sex), hearing variables (hearing thresholds with PTA and HFA), and variables related to the rheumatologic disease (activity, duration, and immunologic analysis). Disease activity is measured as active or nonactive.

### 2.1. Hearing Examination

The first otological test included an initial otoscopy and a hearing screening with PTA and HFA.

All the subjects suitable for inclusion were evaluated based on a questionnaire (Annex A in ISO 389-9: 2009) according to a written protocol based on ISO 389-9 recommendations on the determination of reference hearing threshold levels.

Audiometric tests were conducted by experienced audiometric technicians. For each test frequency, the signal was manually increased by steps of 5 dB until the test person responded, after which the signal was decreased by 10 dB and increased by 5 dB until response. The intensity to which the listener responded three out of five times was recorded as threshold (masking was used). The pure tone hearing thresholds (125–8000 Hz) were measured with a manual audiometer (Madsen Orbiter 922, version 2; Madsen Electronics, Taastrup, Denmark) and equipped with TDH-39 supra-aural earphones (Telephonics Co., Farmingdale, USA). Bone and air conduction audiometry were performed.

HFA thresholds (9, 10, 11.2, 12.5, 14, 16, and 18 kHz) were determined using a Madsen clinical audiometer (Madsen Orbiter 922, version 2; Madsen Electronics, Taastrup, Denmark) with a Sennheiser HDA 200 closed circumaural earphone (Sennheiser Co., Germany). All testing equipment for audiometry was calibrated according to ISO 389-5 (International Organization for Standardization, 2006) and the manufacturer's recommendations. The threshold was defined as the lowest decibel hearing level at which responses occurred in at least 50% of a series of ascending trials (ANSI, 2004) [26, 27].

The limit for the hearing loss to be considered was established when one or more frequencies have higher threshold than the frequency previously identified as normal in the control population [28].

### 2.2. Laboratory Analysis

An immunological analysis of all the parameters, including immunological RA activity (CRP and ESR), subgroup markers of RA (RF and antinuclear antibodies), acute phase reactants (APR), serum cryoglobulins, and anti-cardiolipin (aCL) and antithyroid antibodies, was performed.

### 2.3. Ethics

All the procedures used in this study and practiced on each patient were in agreement and regulated with approval from the “Severo Ochoa” Hospital (Madrid) Institutional Review Board and ethical code identified by the MINUTE: 11/09, 25/11/2009, IEC (Independent Ethics Committee) Internal code: 431-A (65/09), and the Declaration of Helsinki (1983). Informed consent was obtained from each participant. The same person performed all interviews. The subjects received no monetary compensation. Informed consent was obtained from all subjects, after being informed of details about the purpose of the study, according to the recommendations from the Ethics Committee in our centre.

### 2.4. Statistical Analysis

Statistical analyses were performed using SPSS 15 (*Statistical Package for the Social Sciences*) for Windows. This study used quantitative and qualitative descriptive statistics, Chi-square test, Student's* t*-test, and Mann-Whitney* U* test. Logistic regression analysis was achieved by adjusting the estimate for some risk factors, including independent variables such as risk factors related to the patient, subjective hearing or rheumatic disease, with a dependent variable represented by the presence of sensorineural hearing loss. The estimate was established with 95% confidence intervals of the hearing loss prevalence in the PTA (threshold > 30 dB HL) and in the HFA (according to the hearing threshold of the control population for each frequency), adjusted for age (20–29, 30–39, 40–49, and 50–60 age ranges) and sex. A *p* value of <0.05 was considered significant.

## 3. Results and Discussion

The average age of the patients with RA was 50.5 years (interquartile range: 38–59 years), and the patients included 14 males and 39 females with a 26.4%/73.6% male/female ratio. The control population included 71 subjects (142 ears) belonging to Madrid Area 9; the patients were consecutively enrolled, with an average age of 38 years (20–60 years, 50 females and 21 males).

### 3.1. Pure Tone Audiometry

In this study, 43% of the study population with PTA had hearing loss (46 ears). The type of hearing loss was sensorineural, with high-frequency loss predominant. High frequencies were affected in 56.6%, medium frequencies in 17%, and low frequencies in 13.2% of the population. There was a significant difference in hearing thresholds (arithmetic mean) at 6000 Hz (*p* = 0.001) and 8000 Hz (*p* = 0.001) frequencies between patients with RA and the control population.

**Figure 1:**
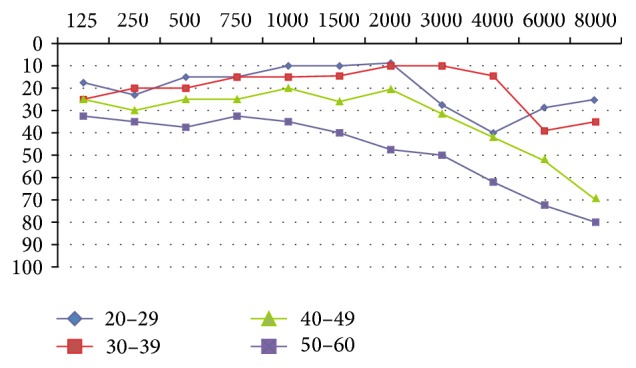
Hearing threshold with pure tone audiometry according to the age range of the study population with RA. Hearing thresholds < 30 dB: hearing normality values with PTA. Arithmetic means (*y*-axis: intensity, dB; *x*-axis: frequency, Hertz or Hz). RA: rheumatoid arthritis. PTA: pure tone audiometry.

SNHL was bilateral and symmetric in 74% of the cases. There were no cases of conductive or mixed hearing loss. Our results showed that patients with RA had greater high-frequency loss than the control population (according to age range) (Figure 1). Regarding sex, there was a significant difference in the 4000 (*p* = 0.038), 6000 (*p* = 0.004), and 8000 Hz (*p* = 0.009) frequencies; there were lower auditory hearing thresholds in females compared to males. We observed that in patients with RA there was a statistically significant relationship between the hearing loss diagnosed by PTA and sex (OR = 3.46 (1.29–9.27)) and the age at which it was diagnosed (OR = 1.09 (1.04–1.15)).

### 3.2. Extended-High-Frequency Audiometry

**Figure 2:**
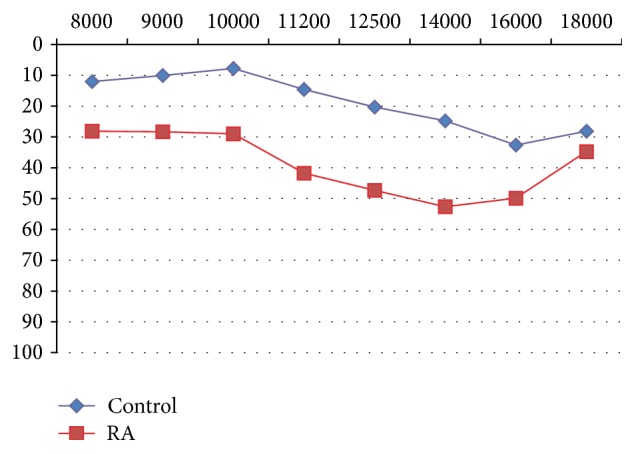
Hearing threshold with ultra high-frequency audiometry, without an age range, in both the control population and the study population with RA. Arithmetic means (*y*-axis: intensity, dB; *x*-axis: frequency, Hertz or Hz). RA: rheumatoid arthritis.

**Figure 3:**
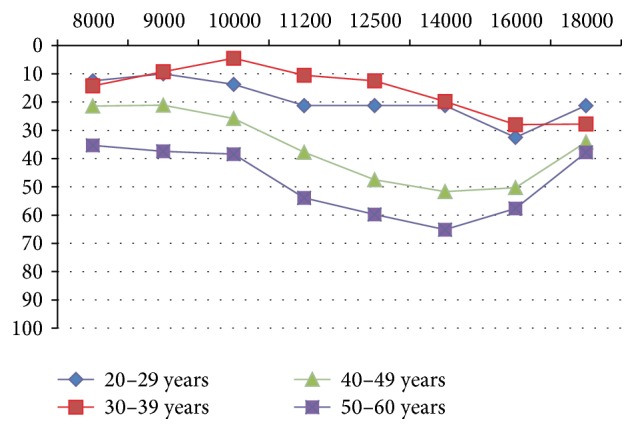
Hearing threshold with ultra high-frequency audiometry, within the age range of the study population with RA. Arithmetic means (*y*-axis: intensity, dB; *x*-axis: frequency, Hertz or Hz). RA: rheumatoid arthritis.

A 69.8% prevalence of high-frequency SNHL in patients with RA was diagnosed using HFA. The arithmetic means of the hearing thresholds in patients with RA were compared with the controls and the result was a significant difference in all frequencies from 8000 to 18000 Hz (*p* < 0.0001) (Figure 2). These results showed that hearing declined in patients with RA in all age ranges, which predominantly included 40-year-old and older patients (Figure 3).

Regarding sex, we obtained a significant difference between the means of the 8000 Hz (*p* = 0.009) and the 10000 Hz (*p* = 0.026) threshold frequencies, between male and female patients with RA, which demonstrated a greater hearing loss in males than in females.

#### 3.2.1. Immunological Study

Regarding the characteristics of the study population, we observed that patients with RA showed a significant relationship between the SNHL measured with HFA and the aCL positivity (OR = 0.38 (0.15–0.94)).

#### 3.2.2. Comparison between PTA and HFA

The diagnosis of SNHL in RA reached 69.8% with HFA and 43.4% with PTA. The chance of diagnosing hearing loss with HFA is 33.6 times higher compared to PTA OR = 29 (4.8–1184.43).

RA may cause SNHL [29], similar to other autoimmune diseases [13, 30]. Although the etiology of SNHL in RA is unknown, there have been a number of physiopathological hypotheses, such as the presence of vasculitis, neuritis, or immune-complexes that deposit in the inner ear or the effect of the possible ototoxic treatment used in the treatment of RA [5, 7, 12, 21, 22, 25].

Nevertheless, nowadays the treatment used in RA includes immune-modulating drugs that could control the progression of the SNHL in some patients. Thereby an early diagnosis of a possible subclinical SNHL by means of HFA (detected just before treatment is applied or along a possible long-term treatment such as chemotherapy) in order to allow a prior treatment (transtympanic or systemic corticosteroids) is of paramount importance.

*(1) PTA in RA*. The prevalence of SNHL with PTA in our study (43.4%) is within the same range as described in previous literature, which has been estimated between 25.2 to 60% [7, 8, 12, 13]. Unlike other studies, we did not observe conductive or mixed hearing loss in our study population (Table 1). Regarding the frequency affected, we obtained results that were consistent with several previous studies regarding the damage caused in high frequencies in patients with RA [5, 8, 10, 17, 31, 32]. Nevertheless, our results differed from those in studies performed by Raut et al. [7], who claimed a greater hearing loss in intermediate frequencies of 500 Hz, 1000 Hz, and 2000 Hz, and from Murdin et al. [14], who reported that patients mainly had hearing loss in low and medium frequencies (from 250 to 4000 Hz). Murdin proposed that the damage caused in low frequencies may be due to the association between RA and endolymphatic hydrops (caused by immune-complex deposits), which initially affects low frequencies and subsequently affects the high frequencies.

A comparison between the study and control groups using PTA revealed a significant hearing loss in patients with RA at frequencies of 6000 and 8000 Hz. Our results differ from other authors, who did not find significant differences between the two populations [12, 13].

Moreover, 73.9% of our patients with RA had bilateral, symmetric SNHL, which was a slightly lower result compared to the results obtained by Özcan et al.: 84.6% with bilateral SNHL [8] although these were clearly higher compared to the findings obtained in other studies [7, 12, 21]. In our study we observed a significant relationship between SNHL (diagnosed using PTA) and age (OR > 1), with a higher risk of developing hearing loss for patients over 40 years of age. In patients older than 50 and who only had unilateral SNHL the hearing thresholds using PTA became worse along the years, similar to findings reported by Halligan's studies [13]. However, studies performed by author did not show a correlation between SNHL and the age of the patient with RA [12, 21, 33].

In our study, we observed a significant relationship between SNHL and sex (OR > 1) with males demonstrating a higher risk of hearing loss and significant differences in high frequencies at approximately 4000, 6000, and 8000 Hz. These findings were consistent with those observed by Dikici et al. [21]. This hearing loss was not related to the greater exposure to noise in males in their working environment, which was an excluding factor in our study.

The persistent findings of high-frequency thresholds in female subjects might be explained due to the fact that the female ear canal has smaller average length and volume. Nevertheless, there could be other explanations for the gender difference, such as differences in exposure to workplace or extracurricular activity noise or different hearing levels as a result of a different phylogenetic origin. Moreover, the aging process may be different in males and females. There are many health factors that influence hearing status (smoking, hypertension, and diabetes) which may also influence the differences observed between males and females [26].

*(2) HFA in RA*. In most of the studies, the hearing loss diagnosis in patients with RA was performed using pure tone audiometry. In few studies a hearing screening was performed using frequencies higher than 8000 Hz [14, 21, 34, 35]. The usefulness of extended-high-frequency audiometry in subclinical diagnosis of hearing loss has been proven [21, 35] and, for this reason, the use of this test from the beginning of the rheumatic disease can provide information on the development of hearing loss, thereby increasing the possibilities of early treatment and prevention.

Furthermore, 69.8% of our patients with RA were diagnosed with SNHL using HFA, which is a higher percentage compared to those obtained using PTA. Some studies showed that there were no references regarding the index of SNHL using HFA, because previous results were global (125–16000 Hz) [21, 36].

Our results do not show a significant correlation between SNHL diagnosed using HFA and age. This finding takes into consideration that normal age-related hearing loss in patients older than 50 from the control population is expected to have higher thresholds than those with RA since hearing loss is generally observed since early stages of the disease [21]. Regarding sex, our findings were consistent with those obtained by Dikici et al. [21] in which a greater hearing loss was observed in males, with significant differences in high-pitched frequencies of 8000 Hz and 10000 Hz.

In our study there were statistically significant differences in all of the frequencies studied between the hearing threshold in both the control population and patients with RA. The hearing loss is greater starting at 40 years of age with thresholds higher than 60 dB.

Öztürk et al. obtained similar results and found significant differences between the control population and the patients with RA, from a frequency 220 to 16000 Hz [36]. In our study, there were minimal hearing changes at 18000 Hz compared to controls. We are not able to compare these results with other studies because a frequency of 18000 Hz was not assessed. Normal age-related hearing loss in patients older than 50 from the control population is expected to have higher thresholds than those with RA since hearing loss is generally observed since early stages of the disease [21].

We found no correlation between SNHL diagnosed using PTA and HFA and the duration of the disease, which was consistent with other studies [12, 14, 32, 33]. However, we did obtain a correlation with the rheumatic disease activity in patients with RA and SNHL, similar to other authors [5, 12, 14, 31, 37]. These parameters were, as mentioned above, immunological RA activity (CRP and ESR), subgroup markers of RA (RF and antinuclear antibodies), acute phase reactants (APR), serum cryoglobulins, and anti-cardiolipin (aCL) and antithyroid antibodies.

The analysis of immunological parameters using specific laboratory tests was performed without obtaining conclusive results [5, 11, 23, 38, 39]. A statistically significant relationship (*p* = 0.029) between SNHL diagnosed using HFA and anti-cardiolipin (aCL) positivity (OR < 1) was observed. This finding might be due to the potential for inner ear thrombosis to be developed, which is found to be associated with high levels of aCL (risk factor for SNHL). In our study, 43.3% of the patients with RA had aCL positivity, which may cause confusion since it is usually associated with SLE [40–42].

No correlation between positivity to anti-nuclear antibodies (ANA), rheumatoid factor (RF), Sjögren's syndrome (SS), aCL, ESR, and SNHL has been reported [5, 12, 14].

## 4. Conclusion

There was a higher rate of patients diagnosed with SNHL using HFA (with the study finding an SNHL prevalence of 69.8%) compared to studies previously reported. SNHL in RA was bilateral and predominantly symmetric, affecting mainly high frequencies. Males over 40 had a greater risk of suffering from SNHL.

There were more cases of subclinical hearing loss diagnosed using HFA compared to PTA. These results lead us to consider HFA as a necessary test to diagnose subclinical hearing loss in patients with RA. An early intervention in this subgroup of patients may cease the development of hearing loss. Thus, we propose that HFA should be performed as a routine screening not only for the study of RA but also for all of the inner ear disorders [26, 28, 43]. This should be taken into consideration not only before a treatment which may cause possible inner ear damage is performed but also along a long-term treatment such as the one received in patients with RA. We recommend a yearly HFA to be performed in patients with RA and other autoimmune diseases.

## Figures and Tables

**Table 1:** Comparative analysis of results of SNHL and CHL as diagnosed using PTA in patients with RA, according to the reviewed sources. SNHL: sensorineural hearing loss; CHL: conductive hearing loss; PTA: pure tone audiometry; RA: rheumatoid arthritis.

Authors	Number of patients	% SNHL (PTA)	% CHL (PTA)
Our study	53	43.4	—
Magaro et al. [5]	20	55	—
Kastanioudakjs et al. [12]	45	35.5	—
Raut et al. [7]	35	60	—
Özcan et al. [8]	37	35.1	24.3
Takatsu et al. [10]	37	36.1	—
Bhama et al. [9]	25	60	12
Halligan et al. [13]	29	40	10
Murdin et al. [14]	55	29.6	1.9
Dikici et al. [21]	20	45	—
Baradaranfar and Doosti [32]	50	60	—

## References

[1] McCabe B. F. (1979). Autoimmune sensorineural hearing loss. *Annals of Otology, Rhinology and Laryngology*.

[2] McCabe B. F. (2004). Autoimmune sensorineural hearing loss. 1979. *Annals of Otology, Rhinology & Laryngology*.

[3] McCabe B. F. (2007). Autoimmune sensorineural hearing loss. *The Annals of Otology, Rhinology, and Laryngology*.

[4] Reiter D., Konkle D. F., Myers A. R., Schimmer B., Sugar J. O. (1980). Middle ear immittance in rheumatoid arthritis. *Archives of Otolaryngology*.

[5] Magaro M., Zoli A., Altomonte L. (1990). Sensorineural hearing loss in rheumatoid arthritis. *Clinical and Experimental Rheumatology*.

[6] Kakani R. S., Mehra Y. N., Deodhar S. D., Mann S. B. S., Mehta S. (1990). Audiovestibular functions in rheumatoid arthritis. *Journal of Otolaryngology*.

[7] Raut V. V., Cullen J., Cathers G. (2001). Hearing loss in rheumatoid arthritis. *Journal of Otolaryngology*.

[8] Özcan M., Karakuş F. M., Gündüz O., Tuncel Ã., Şahin H. (2002). Hearing loss and middle ear involvement in rheumatoid arthritis. *Rheumatology International*.

[9] Bhama M., Bhama L., Agarwal S., Soni N. K. (2005). Auditory functions in rheumatoid arthritis. *Indian Journal of Otology*.

[10] Takatsu M., Higaki M., Kinoshita H., Mizushima Y., Koizuka I. (2005). Ear involvement in patients with rheumatoid arthritis. *Otology and Neurotology*.

[11] García Callejo F. J., Conill Tobías N., Munoz Fernández N., de Paula Vernetta C., Alonso Castañeira I., Marco Algarra J. (2007). Hearing impairment in patients with rheumatoid arthritis. *Acta Otorrinolaringologica Espanola*.

[12] Kastanioudakjs I., Skevas A., Danielidis V., Tsiakou E., Drosos A. A., Moustopoulos M. H. (1995). Inner ear involvement in rheumatoid arthritis: a prospective clinical study. *Journal of Laryngology and Otology*.

[13] Halligan C. S., Bauch C. D., Brey R. H. (2006). Hearing loss in rheumatoid arthritis. *Laryngoscope*.

[14] Murdin L., Patel S., Walmsley J., Yeoh L. H. (2008). Hearing difficulties are common in patients with rheumatoid arthritis. *Clinical Rheumatology*.

[15] Doig J. A., Whaley K., Dick W. C., Nuki G., Williamson J., Buchanan W. W. (1971). Otolaryngological aspects of Sjögren's syndrome. *British Medical Journal*.

[16] Moffat D. A., Ramsden R. T., Rosenberg J. N., Booth J. B., Gibson W. P. (1977). Otoadmittance measurements in patients with rheumatoid arthritis. *Journal of Laryngology and Otology*.

[17] Gussen R. (1977). Atypical ossicle joint lesions in rheumatoid arthritis with sicca syndrome (Sjogren syndrome). *Archives of Otolaryngology*.

[18] Biasi D., Fiorino F., Carletto A., Caramaschi P., Zeminian S., Bambara L. M. (1996). Middle ear function in rheumatoid arthritis: a multiple frequency tympanometric study. *Clinical and Experimental Rheumatology*.

[19] Colletti V., Fiorino F. G., Bruni L., Biasi D. (1997). Middle ear mechanics in subjects with rheumatoid arthritis. *Audiology*.

[20] Frade C., Martin C. (1998). Diagnostic value of the multifrequency tympanometry in active rheumatoid arthritis. *Auris Nasus Larynx*.

[21] Dikici O., Muluk N. B., Tosun A. K., Ünlüsoy I. (2009). Subjective audiological tests and transient evoked otoacoustic emissions in patients with rheumatoid arthritis: analysis of the factors affecting hearing levels. *European Archives of Oto-Rhino-Laryngology*.

[22] Seçkin Ü., Özoran K., Ikinciogullari A., Borman P., Bostan E. E. (2000). Hydroxychloroquine ototoxicity in a patient with rheumatoid arthritis. *Rheumatology International*.

[23] García Berrocal J. R., Ramírez-Camacho R., Vargas J. A., Millan I. (2002). Does the serological testing really play a role in the diagnosis immune-mediated inner ear disease?. *Acta Oto-Laryngologica*.

[24] García-Berrocal J. R., Trinidad A., de la Fuente R., Ramírez-Camacho R., Zurita M., Lobo D. (2004). Controversies and criticisms on designs for experimental autoimmune labyrinthitis. *Annals of Otology, Rhinology and Laryngology*.

[25] Walek H., Fritze W., Kolarz G. (1980). Possible involvement of the auditory system in rheumatoid arthritis. *Zeitschrift fur Rheumatologie*.

[26] Rodriguez Valiente A., Trinidad A., García Berrocal J. R., Górriz C., Ramírez Camacho R. (2014). Extended high-frequency (9–20 kHz) audiometry reference thresholds in 645 healthy subjects. *International Journal of Audiology*.

[27] Rodriguez-Valiente A. R., Fidalgo A. R., García-Berrocal J. R., Camacho R. (2015). Hearing threshold levels for an otologically screened population in Spain. *International Journal of Audiology*.

[28] Debas J., Domingues S. (2002). Patrones de normalidad para la audiometría tonal de altas frecuencias. *Foni Audiológica*.

[29] Worth T. H., Liyanage S. P., Liyanage S. P. (1972). A pilot survey of hearing loss in patients with rheumatoid arthritis. *Scandinavian Journal of Rheumatology*.

[30] Kastanioudakis I., Ziavra N., Voulgari P. V., Exarchakos G., Skevas A., Drosos A. A. (2002). Ear involvement in systemic lupus erythematosus patients: a comparative study. *Journal of Laryngology and Otology*.

[31] Elwany S., El Garf A., Kamel T. (1986). Hearing and middle ear function in rheumatoid arthritis. *The Journal of Rheumatology*.

[32] Baradaranfar M. H., Doosti A. (2010). A Survey of relationship between rheumatoid arthritis and hearing disorders. *Acta Medica Iranica*.

[33] Salvinelli F., Cancilleri F., Casale M. (2004). Hearing thresholds in patients affected by rheumatoid arthritis. *Clinical Otolaryngology and Allied Sciences*.

[34] Karabulut H., Dagli M., Ates A., Karaaslan Y. (2010). Results for audiology and distortion product and transient evoked otoacoustic emissions in patients with systemic lupus erythematosus. *Journal of Laryngology and Otology*.

[35] Maciaszczyk K., Durko T., Waszczykowska E., Erkiert-Polguj A., Pajor A. (2011). Auditory function in patients with systemic lupus erythematosus. *Auris Nasus Larynx*.

[36] Öztürk A., Yalçin Ş., Kaygusuz İ. (2004). High-frequency hearing loss and middle ear involvement in rheumatoid arthritis. *American Journal of Otolaryngology*.

[37] Goodwill C. J., Lord I. J., Jones R. P. (1972). Hearing in rheumatoid arthritis. A clinical and audiometric survey. *Annals of the Rheumatic Diseases*.

[38] García Callejo F. J., Corts J., de Paula Vernetta C., Laporta P., Ramírez Sabio J., Marco Algarra J. (2006). Cormobidity of sensorineural hearing loss and other autoimmune diseases. Usefulness of laboratory tests. *Acta Otorrinolaringologica Espanola*.

[39] Lobo D., López F. G., García-Berrocal J. R., Ramírez-Camacho R. (2008). Diagnostic tests for immunomediated hearing loss: a systematic review. *Journal of Laryngology and Otology*.

[40] Tumiati B., Casoli P. (1995). Sudden sensorineural hearing loss and anticardiolipin antibody. *American Journal of Otolaryngology—Head and Neck Medicine and Surgery*.

[41] Hisashi K., Komune S., Taira T., Uemura T., Sadoshima S., Tsuda H. (1993). Anticardiolipin antibody-induced sudden profound sensorineural hearing loss. *American Journal of Otolaryngology—Head and Neck Medicine and Surgery*.

[42] Green L., Miller E. B. (2001). Sudden sensorineural hearing loss as a first manifestation of systemic lupus erythematosus: association with anticardiolipin antibodies. *Clinical Rheumatology*.

[43] Rodríguez Valiente A., Roldán Fidalgo A., Villarreal I. M., García Berrocal J. R. (2015). Extended high-frequency audiometry (9,000–20,000 hz). Usefulness in audiological diagnosis. *Acta Otorrinolaringológica Española*.

